# Universal Template-Assisted, Cloning-free Method for the Generation of Small RNA-Expressing Dumbbell-Shaped DNA Vectors

**DOI:** 10.1016/j.omtm.2019.08.008

**Published:** 2019-08-31

**Authors:** Samantha Leeanne Cyrill, Avantika Ghosh, Pei She Loh, Genim Siu Xian Tan, Volker Patzel

**Affiliations:** 1 Department of Microbiology & Immunology, Yong Loo Lin School of Medicine, National University of Singapore, Block MD4, Level 5, 5 Science Drive 2, Singapore 117545, Singapore; 2 Department of Medicine, Addenbrooke’s Hospital, University of Cambridge, Cambridge CB2 0QQ, UK

**Keywords:** dumbbell vector, DNA minimal vector, cloning-free generation, small RNA expression, high-throughput-compatible vector production, functional genomics, gene knockdown in primary cells, small hairpin RNA, microRNA

## Abstract

Dumbbell-shaped DNA minimal vectors represent genetic vectors solely composed of the gene expression cassette of interest and terminal closing loop structures. Dumbbell vectors for small hairpin RNA or microRNA expression are extremely small-sized, which is advantageous with regard to cellular delivery and nuclear diffusion. Conventional strategies for the generation of small RNA-expressing dumbbell vectors require cloning of a respective plasmid vector, which is subsequently used for dumbbell production. Here, we present a novel cloning-free method for the generation of small RNA-expressing dumbbell vectors that also does not require any restriction endonucleases. This new PCR-based method uses a universal DNA template comprising an inverted repeat of the minimal H1 promoter and the miR-30 stem. The sequences coding for small RNA expression are introduced by the PCR primers. Dumbbells are formed by denaturing and reannealing of the PCR product and are covalently closed using ssDNA ligase. The new protocol generates plus- and/or minus-strand dumbbells, both of which were shown to trigger efficient target gene knockdown. This method enables fast, cheap production of small RNA-expressing dumbbell vectors in a high throughput-compatible manner for functional genomics screens or, as dumbbells are not prone to transgene silencing, for knockdown studies in primary cells.

## Graphical Abstract

**Figure:**
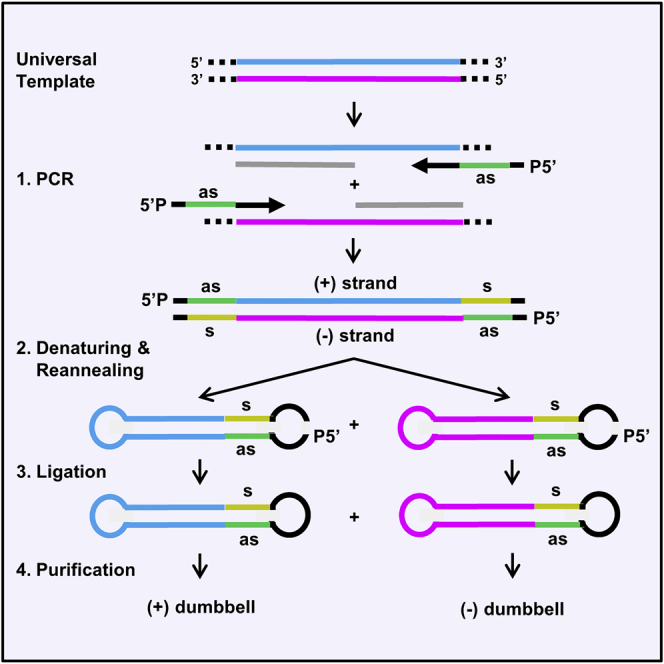

## Introduction

Small or short hairpin RNAs (shRNAs) are artificial hairpin-structured RNAs that can endogenously be transcribed from recombinant genes to efficiently trigger RNAi. For shRNA gene delivery, researchers explore viral or non-viral delivery vectors. While viral vectors are costly and often trigger immune responses or pose the risk of genomic vector integration, many non-viral delivery vectors involve non-nucleic acid helper functions that can be toxic to the cells.^1^, ^2^ The simplest non-viral vectors are naked DNA-based vector systems, three different types of which have been described so far: plasmids, DNA minicircles, and dumbbell-shaped DNA minimal vectors. Whereas plasmid-based gene expression is rapidly silenced in primary cells and *in vivo*, minicircles and dumbbell vectors do not suffer from transgene silencing and have shown promising results in preclinical and clinical trials.^3^, ^4^, ^5^, ^6^, ^7^ However, compared to minicircles, which require a minimum size of 300 bp due to circular tension,^8^ dumbbell vectors have no lower size limit and can virtually be as short as the shRNA gene. The small dumbbell size, in combination with its linear structure, was shown to facilitate cellular delivery and, in particular, nuclear vector diffusion.^9^ Four methods have been reported for the generation of shRNA-expressing dumbbell vectors: First, enzymatic ligation assisted by nucleases (ELAN), a protocol in which intermolecular dumbbell ligation is supported by endonucleolytic cleavage of misligated off-pathway products;^10^ second, a protocol in which the expression cassette is amplified by PCR followed by nicking enzyme cleavage to produce 5′ overhangs which then form the dumbbell loops in an intramolecular ligation;^11^, ^12^ third, a method that combines features of the first two protocols generating size-minimized hairpin template-transcribing shRNA-expressing dumbbell vectors;^9^, ^13^ and finally, a gap-primer PCR-based method that employs chemically modified primers and an intramolecular ligation for the efficient generation of superior dumbbell vectors that are characterized by internal loops and improved nuclear targeting activities.^14^ In general, protocols forming the dumbbell structure during an intramolecular ligation reaction exhibit highest vector yields. In order to generate dumbbell vectors for the expression of novel shRNAs, all of the above protocols depend on a cloning step and/or require endonucleases.

Here, we report a cloning-free method for the generation of shRNA-expressing dumbbell vectors. This PCR-based method uses a universal template and sequences coding for a specific shRNA are introduced by the PCR primers. This novel protocol produces size-minimized hairpin-template transcribing dumbbells, does not require any restriction or nicking endonucleases, and is high throughput compatible.

## Results

### Universal Template-Assisted, Cloning-free Method for the Generation of shRNA-Expressing Dumbbell Vectors

**Figure 1:**
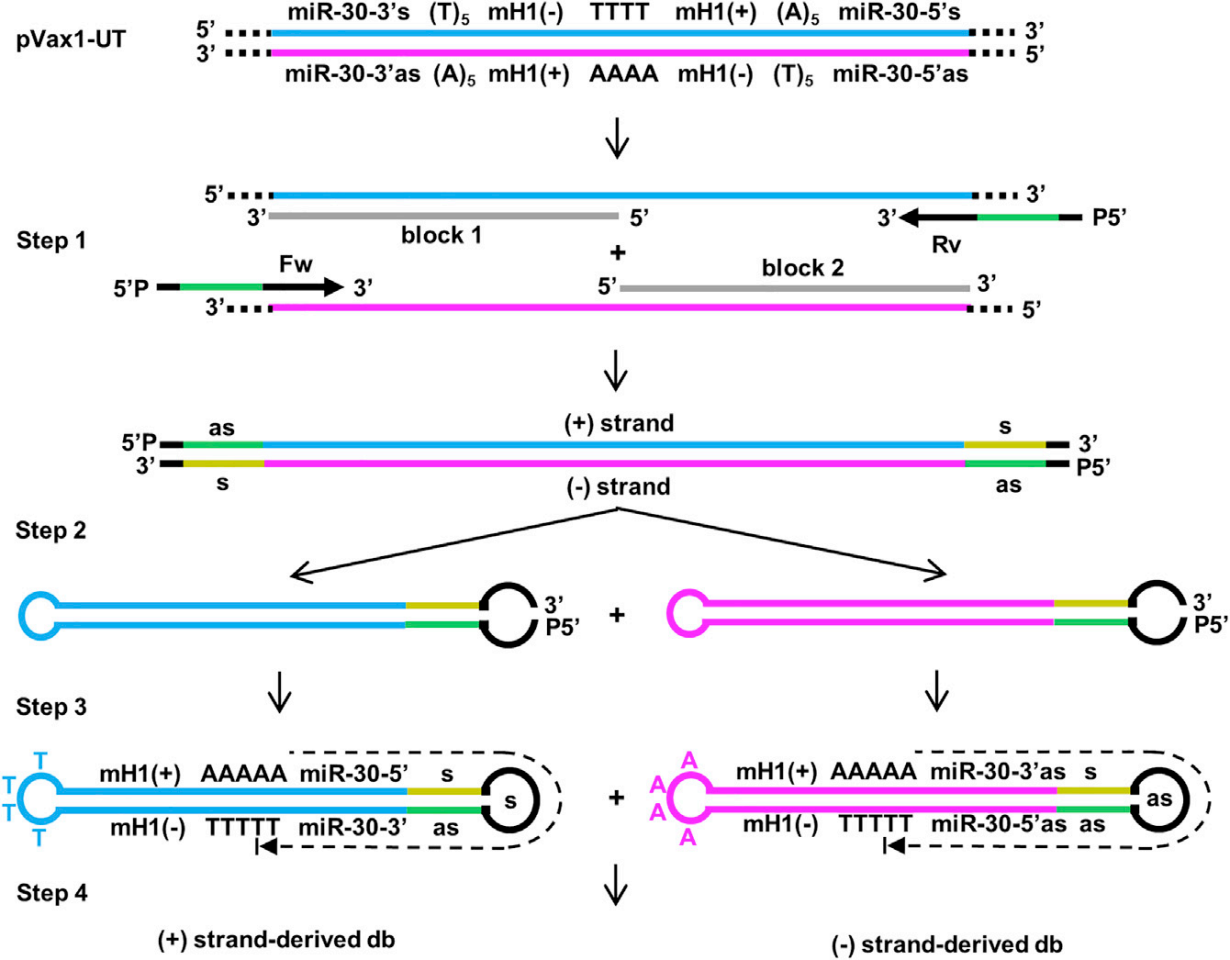
Scheme for Universal Template (UT)-Assisted Cloning-free Dumbbell Production The universal DNA template is a 262-bp double-stranded DNA that comprises an inverted repeat of the 99-bp minimal H1 promoter (mH1), a polymerase III transcriptional terminator (T_5_), and the hsa-miR-30 precursor stem. The inverted repeats are separated by four T’s (plus strand). The protocol for dumbbell generation and purification comprises four steps. Step 1: PCR amplification of the universal template using 5′ phosphorylated forward (Fw) and/or reverse (Rv) primers, both introducing either the antisense or sense strand of a shRNA together with half of the shRNA loop sequence; blocking oligos block 1 and block 2 are added into the reaction to suppress template refolding and to facilitate primer binding. Dotted lines represent plasmid sequences beyond the UT. Step 2: for dumbbell structure prefolding, the PCR product is diluted, heat-denatured, and slowly cooled down to room temperature. Step 3: dumbbell structures are covalently closed using a single-strand DNA ligase. Step 4: treatment with T7 DNA polymerase removes oligos and non-ligated dumbbell DNA, yielding covalently closed dumbbell vector DNA. Dotted arrows indicate transcribed sequences. All steps, cyan, (+) strand UT DNA; magenta, (−) minus strand UT DNA; gray, blocking oligos; green, shRNA sense (s) sequence; yellow, shRNA antisense (as) sequence.

Recently, we reported the design of minimized hairpin template-transcribing dumbbell vectors.^13^ In these vectors, redundant sequences of linear shRNA or pre-microRNA (miRNA) expression cassettes were eliminated and transcription goes around one of the dumbbell loops. This novel dumbbell design facilitates the development of a novel cloning-free method for the generation of such vectors, which is described here (Figure 1). The new method is based on PCR amplification of a universal DNA template which comprises an inverted repeat of (1) the minimal H1 promoter,^15^ (2) a polymerase III transcriptional terminator (T_5_), and (3) the hsa-miR-30 precursor stem (Figure S1A). The hsa-mir-30 stem was reported to facilitate shRNA processing and has been successfully implemented in dumbbell vector design.^13^, ^16^ Once generated, the universal template can be used for cloning-free generation of any shRNA-expressing dumbbell vectors. Sequences coding for the expression of the respective small RNA are introduced during the PCR by the PCR primers (step 1). Irrespective of the small RNA-specific 5′ portion of the PCR primers, they all harbor the same 3′ terminal target binding sites which facilitates parallelized PCR amplifications. Both strands of the universal DNA template have a high degree of self-complementarity, and to improve its amplification, blocking oligos are added to the PCR reaction to suppress intramolecular refolding of the denatured DNA and to support primer binding. Each of the two DNA strands (+ and −) of the resulting double-stranded PCR product yields, after dilution, heat denaturation, and intramolecular refolding, an open dumbbell scaffold with dangling 5′ and 3′ ends (step 2). These ends are then ligated using a single-stranded DNA (ssDNA) ligase (step 3). All DNA molecules harboring 5′ or 3′ ends are removed by exonuclease digestion yielding clean, covalently closed dumbbell vectors (step 4). With the decision of using either one or two 5′-phosphorylated PCR primers, the plus strand, the minus strand, or both strands will produce dumbbell vectors.

### Generation of a Universal PCR Template

Generation of the universal template was challenging due to the high degree of self-complementarity and all attempts to generate the universal template by gene synthesis failed. Instead, the universal template was assembled from two pairs of complementary oligodeoxyribonucleotides (oligos) in which the self-complementary sequence portions were separated from each other (Figures S1A–S1C). Pairs of complementary oligos were annealed, each forming one complementary 3′ overhang and either a *Hind*III or *Bam*HI 5′ overhang. Pairs of annealed oligos were then first ligated using the adhesive 3′ ends, gel purified, and inserted into the cloning vector pVAX1 using the *Hind*III and *Bam*HI cloning sites, yielding the universal template vector pVAX1-UT. Successful cloning of the universal template was proven by analytical restriction endonuclease cleavage and subsequent gel electrophoresis of the fragments as well as by sequencing: The *Hind*III/*Bam*HI double digestion yielded the expected insert size of 262 bp; sequencing of the complete insert was unsuccessful due to insert self-complementarity, but the cloning sites could be sequenced (Figures S1D and S1E).

### PCR Amplification of the Universal Template and Dumbbell Vector Ligation

**Figure 2:**
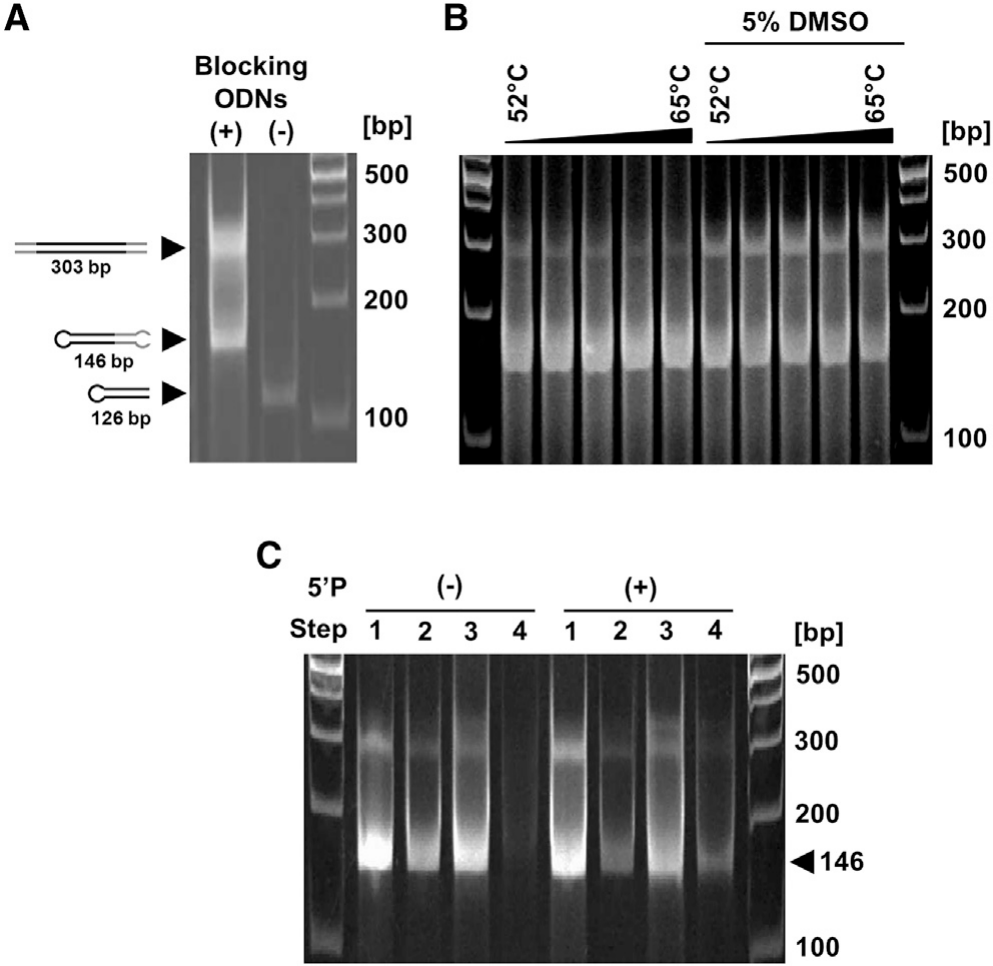
PCR-Based Universal Template (UT)-Assisted Dumbbell Production (A) PCR amplification of the UT depends on the addition of blocking oligos. Without adding blocking oligos (−), no PCR amplification was observed, and only 126 bp refolded UT single-strands were detected. When adding blocking oligos (+), both the PCR-amplified double-stranded dumbbell DNA (303 bp) and refolded dumbbell single strands (146 bp) were detected. (B) Optimization of PCR conditions. PCR yields were virtually independent of the annealing temperature in the range between 52°C and 65°C. Addition of 5% DMSO elevated the yields of double-stranded dumbbell DNA. (C) Assessment of DNA products referring to steps 1 to 4 defined in Figure 1. Step 1: PCR UT amplification yields double-stranded dumbbell DNA (303 bp) and refolded dumbbell single strands (146 bp). Step 2: heat denaturation and refolding converts double-stranded dumbbell DNA into dumbbell single strands. Step 3: single-stranded DNA ligation covalently closes dumbbell vector DNA if 5′ phosphorylated primers were used for PCR. Step 4: exonuclease treatment removes un-ligated DNA and yields covalently closed dumbbell vectors (146 bp).

Next, we aimed to PCR-amplify the universal template using primers that introduced the sequence coding for a published firefly luciferase-targeting shRNA.^9^ However, intrinsic self-complementarity of the universal template was impeding conventional PCR amplification, which did not yield any product of the expected size. Products were observed after adding two long blocking oligos into the PCR reaction (Figure 2A). These blocking oligos were designed such that they were complementary to the respective 5′ half of the plus or the minus strand of the universal template, thus suppressing intramolecular strand refolding and facilitating primer binding to the 3′ ends of the template DNA (Figure 1). Because the blocking oligos bind to the universal template sequence, they represent a constant, target- and shRNA-independent component of this dumbbell generation protocol. The obtained PCR products corresponded in size with the double-stranded universal temple (303 bp) and the refolded single strands (146 bp). Addition of 5% (v/v) DMSO into the PCR reaction yielded more of the larger product, indicating a more efficient amplification as primer binding and extension competed more successfully with single-strand refolding (Figure 2B). Heat denaturation and refolding of the purified PCR products then yielded more of the hairpin structured single-strands (Figure 2C, lanes 2). As expected, the ssDNA ligation (lanes 3) and subsequent exonuclease digestion (lanes 4) yielded exonuclease-resistant dumbbell vector DNA only if 5′-phorsphorylated primers were used for the PCR (Figure 2C).

### Generation of Plus- and/or Minus-Strand-Derived Dumbbell Vectors

**Figure 3:**
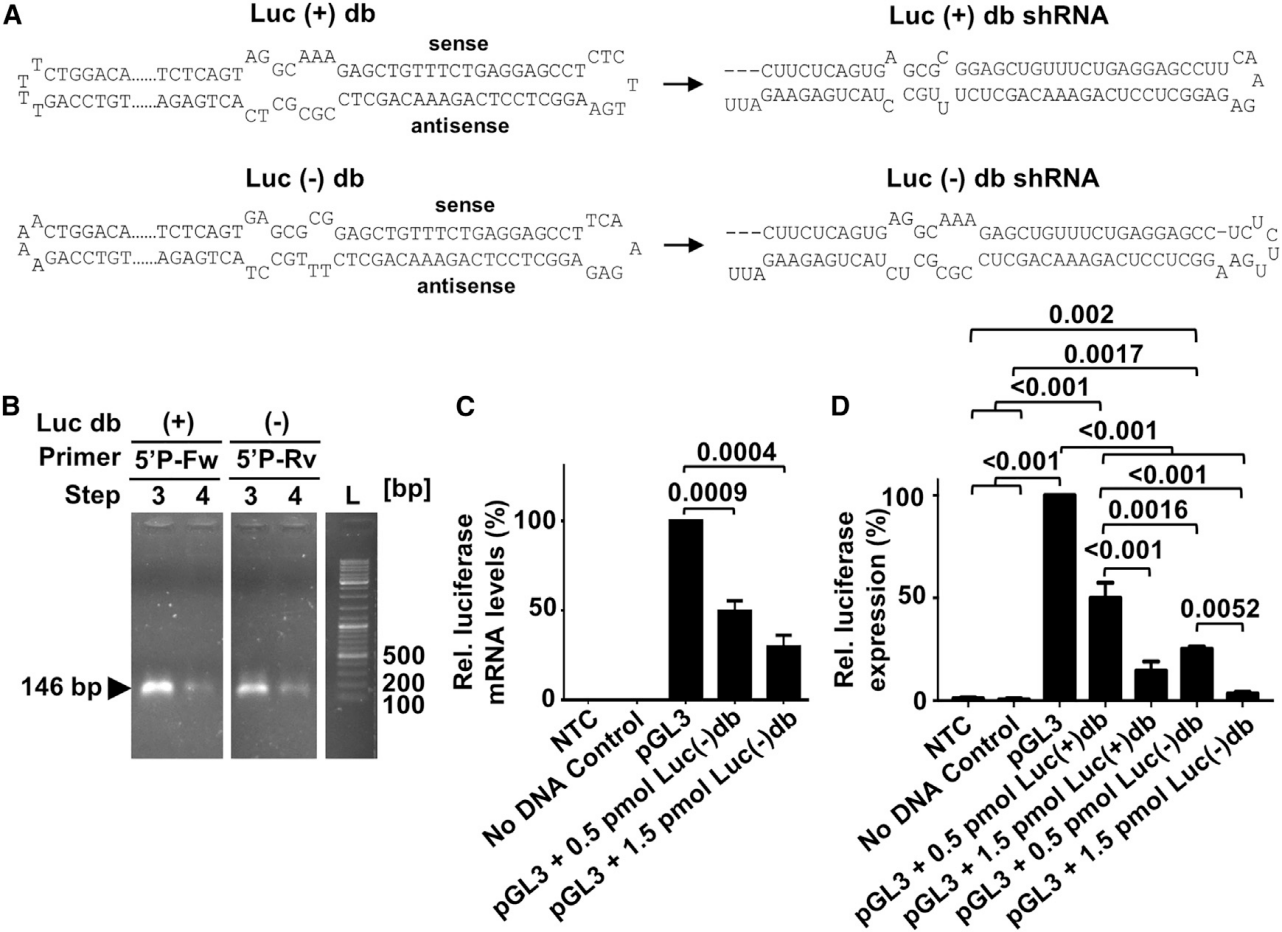
Knockdown of Firefly Luciferase in HEK293T Cells by Plus (+) and Minus (−) Strand-Derived Luciferase-Targeting (Luc) Dumbbell (db) Vectors (A) Sequences and structures of dumbbell vectors and transcribed luciferase-targeting shRNAs. shRNA secondary structures were drawn according to predictions by *mfold* and *RNAfold*. (B) Selective generation of (+) or (−) strand-derived db vectors using either phosphorylated forward (5′P-Fw) or reverse (5′P-Rv) primers. Steps refer to steps 1 to 4 defined in Figure 1. (C and D) Functional validation of plus (+) and/or minus (−) strand-derived luciferase targeting db vectors. Cells were co-transfected with firefly luciferase reporter vector pGL3 and 0.5 or 1.5 pmol dumbbell vector DNA. NTC, no transfection control. Firefly luciferase mRNA (C) or expression (D) levels relative to the uninhibited negative control were measured 48 h post-transfection using qRT-PCR or luciferase reporter assays. Relative RNA levels were calculated in terms of fold change (2^−ΔΔCt^), where ΔCt = C_t luciferase_ − C_t β-actin_. Values are mean values ± SEM of three independent experiments. The statistical analysis was performed using Student’s t test (C) or repeated one-way ANOVA with Tukey’s post hoc multiple comparison test (D).

With the decision to use either a 5′-phosphorylated forward primer, a 5′-phosphorylated reverse primer, or two phosphorylated primers for PCR, only (1) plus-strand-derived dumbbells, (2) minus-strand-derived dumbbells, or (3) a mix of both can be generated (Figures 1 and S2). In order to obtain a mix of plus- and minus-strand-derived dumbbells, it does not make a difference if the 5′ ends of the PCR primers or alternatively of the PCR product are phosphorylated (Figure S3). In this example, plus- and minus-strand-derived dumbbells and the expressed shRNAs are very similar, but not identical, as they differ with regard to sequence and structure in the loops and in the hsa-miR-30 stem (Figures 3A and S2). The asymmetry in the miRNA stem region is owed to the fact that correct transcription of a partly mismatched miRNA precursor RNA can only be achieved if the hairpin template-transcribing dumbbell harbors corresponding mismatches as well. Consequently, only the plus-strand-derived dumbbell expresses the shRNA extended with the original miR-30 stem (Figure 3A). The shRNA expressed from the minus-strand-derived dumbbell is extended with a miR-like stem formed by the antisense sequences of miR-30 and carries a loop that represents the reverse complement of the loop in the plus-strand-derived shRNA. The observed conversion yield, i.e., the fraction of refolded 146-bp dumbbell vector DNA that was successfully ligated and resisted subsequent exonuclease treatment, was measured to be 34% or 28% for the production of the plus- or minus-strand-derived luciferase-targeting dumbbells (Figure S4). Considering that only one PCR primer was phosphorylated for the generation of these dumbbells and that consequently only half of the refolded DNA could theoretically be ligated, then the actual conversion yield of ligatable plus- or minus-strand-derived dumbbell DNA is 68% or 56%. In these reactions, we ligated 6 μg of DNA using 100 U of CircLigase.

### Plus- and Minus-Strand-Derived Dumbbell Vectors Trigger Target Gene Knockdown

**Figure 4:**
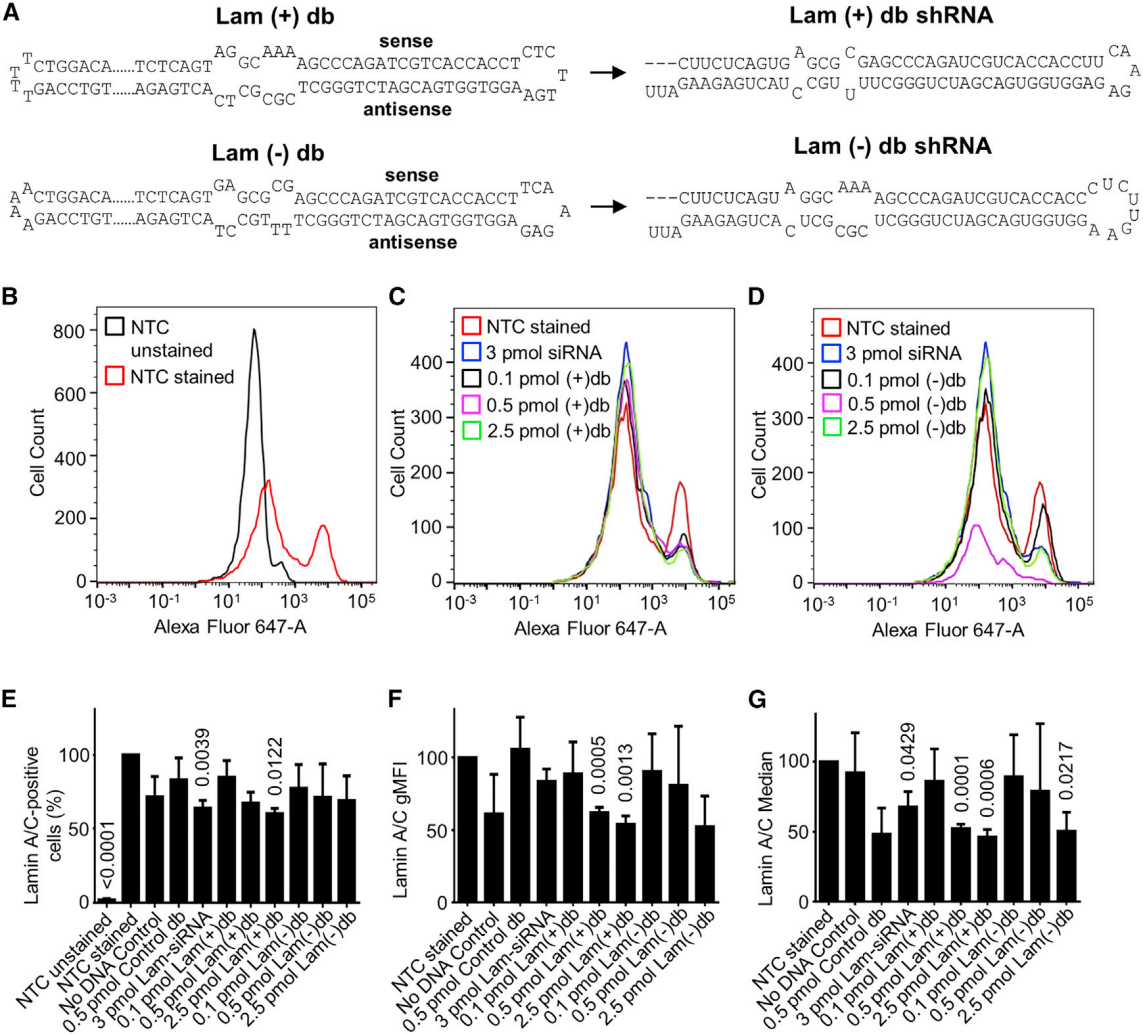
Knockdown of Lamin A/C in HEK293T Cells by Plus (+) and Minus (−) Strand-Derived Lamin-Targeting (Lam) Dumbbell (db) Vectors Monitored Using Intracellular FACS (A) Sequences and structures of dumbbell vectors and transcribed lamin A/C-targeting shRNAs. shRNA secondary structures were drawn according to predictions by *mfold* and *RNAfold*. (B–D) Representative histogram overlays of one experiment. (B) Stained (primary anti-lamin A+C antibody plus secondary donkey anti-rabbit IgG H&Ls) versus unstained (primary anti-lamin A+C antibody only) non-transfected live cells. (C) Knockdown triggered by 0.1, 0.5, or 2.5 pmol plus-strand-derived anti-lamin A/C shRNA-expressing db vectors [Lam(+)db] or 3 pmol anti-lamin A/C positive control siRNA (Lam-siRNA). (D) Knockdown triggered by 0.1, 0.5, or 2.5 pmol minus strand-derived anti-lamin A/C shRNA-expressing db vectors [Lam(−)db] or 3 pmol Lam-siRNA. (E–G) Knockdown of lamin A/C in stained HEK293T cells relative to the non-transfected cells (100%) represented by the fraction of lamin A/C-stained cells (E), the geometric mean fluorescence intensity (gMFI) of lamin A/C-stained cells (F), and the median fluorescence intensity of lamin A/C-stained cells (G). The control dumbbell (control db) was a 1:1 mix of plus- and minus-strand-derived luciferase targeting dumbbell DNA. Values are mean values ± SEM of three independent experiments. The statistical analysis was performed using Student’s t test. p values indicate significance relative to the stained no-transfection control. (B–G) NTC, no transfection control; no DNA control, buffer transfected cells.

Employing the above protocol using either phosphorylated forward or reverse primers, we generated both plus- and minus-strand-derived luciferase- or lamin A/C-targeting dumbbells in separate reactions (Figures 3 and 4). The purity of the vectors after exonuclease treatment was controlled using agarose gel electrophoresis (Figure S5A). Additional capillary gel electrophoresis determined the purity of the minus-strand-derived lamin A/C-targeting dumbbell to be 83% (Figure S5B). To measure dumbbell vector-triggered luciferase knockdown, HEK293T cells were co-transfected with the luciferase expression vector pGL3-Control and 0.5 or 1.5 pmol of plus- or minus-strand-derived dumbbell vector DNA using Lipofectamine 2000. 48 h post-transfection, firefly luciferase mRNA and activity levels were quantified relative to the pGL3-Control vector (Figures 3C and 3D). Both dumbbells triggered a significant, dose-dependent luciferase knockdown, which surprisingly was more pronounced in case of the minus-strand-derived dumbbell vector, indicating the non-natural miR-like stem was functional. The knockdown triggered by the plus-strand-derived dumbbell was 85% (p < 0.001) or 50% (p < 0.001) at 1.5 or 0.5 pmol vector DNA, and the minus-strand-derived dumbbell triggered 97% (p < 0.001) or 75% (p < 0.001) knockdown at 1.5 or 0.5 pmol DNA, respectively, relative to the pGL3 positive control. To investigate the knockdown of lamin A/C, HEK293T cells were transfected with 0.1, 0.5, or 2.5 pmol of plus- or minus-strand-derived dumbbell vector DNA or alternatively with 3 pmol siGENOMELamin A/C positive control small interfering RNA (siRNA) or 0.5 pmol luciferase-targeting dumbbell control vector DNA (1:1 mix of plus- and minus-strand-derived dumbbells) using Lipofectamine 3000. 48 h post transfection, intra-cellular lamin A/C was stained using rabbit anti-lamin A+C primary antibody and donkey anti-rabbit immunoglobulin G (IgG) heavy and light chains (H&Ls) AF647 secondary antibody, and lamin A/C knockdown was monitored by flow cytometry analyses (Figures 4 and S6). While the plus-strand-derived dumbbell triggered a significant, dose-dependent lamin A/C knockdown at 2.5 or 0.5 pmol DNA, the knockdown observed with the minus-strand-derived dumbbell was less pronounced.

## Discussion

The protocol described here combines all the advantages of previously reported protocols for dumbbell vector production. It represents (1) a cloning-free protocol that (2) does not involve any restriction or nicking endonucleases, (3) employs an efficient intra-molecular ligation reaction, and (4) allows production of extremely small hairpin template-transcribing dumbbell vectors. The previously described gap-primer PCR protocol also involves an intra-molecular ligation but requires a cloning step for the generation of every new vector, and it is not suitable to generate hairpin template-transcribing vectors due to the presence of abasic sequence positions.^14^ Conversely, the method described by Jiang et al.^9^ and Jiang and Patzel^13^ is suitable to produce hairpin template-transcribing dumbbells but requires restriction and nicking endonucleases and involves a less efficient inter-molecular ligation reaction. The PCR primers used for the protocol reported here always harbor the same 3′ terminal template binding sites as well as a 5′ terminal sequence that depends on and changes with the respective small RNA but which is to a great extent identical within each respective primer pair. Hence, the primer annealing temperatures are always the same and primer dimer formation can widely be excluded, which both facilitates parallelized PCR reactions using a single cycling program. The subsequent ligation reaction represents an intramolecular ligation that is generally more efficient compared with alternative protocols involving intermolecular loop ligation. As a corollary, the conversion yields observed for this method are higher than those reported for protocols employing inter-molecular ligation reactions. For the gap-primer PCR method, higher conversion yields of up to 92% were observed when ligating double-stranded nicked dumbbell DNA using the T4 DNA ligase; however, only slightly higher conversion yields of 75% were observed with the gap-primer PCR method when ligating dangling single-stranded 5′ ends with base-paired 3′ ends using the CircLigase. The purity of dumbbell DNA produced with the method described here was within the purity range of 82% to 94% of vectors produced with the gap-primer PCR method. Additional purification steps will be required for future preclinical and clinical applications.

We demonstrate the proof-of-principle that this new method can generate partly mismatched shRNA-expressing dumbbell vectors, indicating the technology might also be explored for the generation of miRNA-expressing dumbbells. Mismatches in dumbbell vectors were reported earlier and demonstrated not to impair vector activity.^13^ On the contrary, terminal single-nucleotide mismatches were found to improve nuclear targeting and activity of dumbbell-shaped expression vectors.^14^

We observed that among the luciferase- or lamin A/C-targeting dumbbells, the minus- or plus-strand-derived dumbbell exhibited a stronger target gene knockdown activity, respectively. This difference might be assigned to differences with regard to the efficiency and accuracy of endogenous shRNA processing by Dicer, which depends on the sequence and structure of shRNA loops and stems. Here, we employed the hsa-miR-30 stem, as miRNA stems were reported to facilitate shRNA processing and knockdown activities in most of the cases.^16^ Consequently, though the respective plus- and minus-strand-derived dumbbells code for identical guide sequences, the transcribed small hairpin RNAs comprise different microRNA stems and different loops as emphasized above. Hence, differences in Dicer processing might lead to different guide RNA levels and/or differences with regard to the exact 5′ and 3′ termination of guide RNA sequences. These differences can account for the observation that, depending on the targeted sequences and the corresponding guide RNA sequences and structures, either the plus- or the minus-strand-derived dumbbell triggers stronger target gene knockdown. However, when forgoing the inclusion of a miRNA stem and when concurrently considering palindromic loop sequences, both plus- and minus-strand-derived dumbbells would be identical, and a single reaction would generate a single vector only.

In conclusion, this novel method efficiently generates size-minimized hairpin template-transcribing dumbbells in a short period of time and at low costs and can be explored for the parallelized production of shRNA or miRNA expression vectors for functional genomics screens or drug development.

## Materials and Methods

### Oligodeoxyribonucleotides (ODNs) and Primers

#### Universal Template

Due to internal self-complementarity, the universal template could not be generated by gene synthesis and instead was assembled from two pairs of complementary oligodeoxyribonucleotides (IDT, Skokie, IL, USA) (Figures S1A and S1B): oligo UT1, 5′-**AGCTT**CGCGCTCACTGAGAAGATTTTTCTGTGCTCTCATACAGAACTTATAAGATTCCCAAATCCAAAGACATTTCACGTTTATGGTGATTTCCCAGAACACATAGCGACATGCAAATATGAATTGTCCAGTT-3′; oligo UT2, 5′P-GGACAATTCATATTTGCATGTCGCTATGTGTTCTGGGAAATCACCATAAACGTGAAATGTCTTTGGATTTGGGAATCTTATAAGTTCTGTATGAGAGCACAGAAAAATCTTCTCAGTGAGCGCGA-3′; oligo UT3, 5′P-TTCTGGACAATTCATATTTGCATGTCGCTATGTGTTCTGGGAAATCACCATAAACGTGAAATGTCTTTGGATTTGGGAATCTTATAAGTTCTGTATGAGAGCACAGAAAAATCTTCTCAGTAGGCAAAG-3′; oligo UT4, 5′-**GATCC**TTTGCCTACTGAGAAGATTTTTCTGTGCTCTCATACAGAACTTATAAGATTCCCAAATCCAAAGACATTTCACGTTTATGGTGATTTCCCAGAACACATAGCGACATGCAAATATGAATTGTCCAGAAAACT-3′. Bold indicates *Hind*III and *Bam*HI compatible overhangs; underlined indicates dumbbell loop-forming tetranucleotide. The 5′ phosphorylated oligos UT2 and UT3 were hybridized with the complementary oligos UT1 and UT4, respectively. The resulting UT1/UT2 and UT3/UT4 duplexes were ligated to form the universal template sequence bearing *Hind*III and *Bam*HI-compatible 5′ overhangs, which was subsequently cloned into pVax1 (Thermo Fisher Scientific, Waltham, MA, USA), yielding the universal template vector pVax1-UT (Figure S1B). For cloning we used the recA-deficient *E. coli* strain Top10. Cloning of the universal template was confirmed by PCR and by FastDigest *Bam*HI/*Hind*III (Thermo Fisher Scientific, Waltham, MA, USA) endonucleolytic cleavage followed by analytical agarose gel electrophoresis, which yielded the expected insert size of 262 bp, and by sequencing of the ligation sites (Figures S1C and S1D). Sequencing of the complete universal template was unsuccessful due to the high degree of self-complementarity.

#### Primers for the Production of Firefly Luciferase- or Lamin A/C-Targeting shRNA-Expressing Dumbbells

Luciferase- or lamin A/C-specific primers were synthesized by AITbiotech (Singapore) or IDT (Singapore). Uppercase letters indicate the universal template binding sites, and lowercase letters indicate the shRNA coding sequences in which the loop-forming nucleotides are underlined: forward primers, FP_Luciferase 5′-tgaaggctcctcagaaacagctcCGCGCTCACTGAGAAGATTT-3′; FP_Lamin 5′-tgaaagcccagatcgtcaccacccgcCGCGCTCACTGAGAAGATTT-3′; reverse primers, RP_Luciferase 5′-agagaggctcctcagaaacagctcTTTGCCTACTGAGAAGATTTTTCTGT-3′. RP_Lamin 5′-agagaagcccagatcgtcaccaccttTTTGCCTACTGAGAAGATTTTTCTGT-3′.

#### Blocking ODNs

Two blocking ODNs (IDT, Skokie, IL, USA) were added to the PCR to suppress refolding and self-priming of the universal template strands: Block_1, 5′-GGACAATTCATATTTGCATGTCGCTATGTGTTCTGGGAAATCACCATAAACGTGAAATGTCTTTGGATTTGGGAATCTTATAAGTTCTGTATGAGAGCACAGAAAAATCTTCTCAGTGAGCGCGA-3′; Block_2, 5′-TTCTGGACAATTCATATTTGCATGTCGCTATGTGTTCTGGGAAATCACCATAAACGTGAAATGTCTTTGGATTTGGGAATCTTATAAGTTCTGTATGAGAGCACAGAAAAATCTTCTCAGTAGGCAAAG-3′.

#### Primers for qRT-PCR

Primers for the quantification of luciferase and β-actin mRNA levels were synthesized by AITbiotech (Singapore). PCR forward primers are as follow: qPCR_FP_Luciferase 5′-CGCTGGGCGTTAATCAAAGA-3′; qPCR_RPb-actin 5′-CTGGCACCCAGCACAATG-3′. Reverse transcription and PCR reverse primers are as follows: qPCR_RP_Luciferase 5′-GTGTTCGTCTTCGTCCCAGT-3′; qPCR_RPb-actin 5′- GCCGATCCACACGGAGTACT-3′.

#### Primer Phosphorylation

For the generation of strand-specific dumbbell vectors, either the forward or the reverse primers were 5′-phosphorylated. Each 50 pmol primer was incubated with 10 U T4 polynucleotide kinase (Thermo Fisher Scientific, Waltham, MA, USA) in the presence of 1 mM ATP at 37°C for 20 min followed by heat inactivation of the enzyme at 75°C for 10 min.

### Dumbbell Vector Generation

#### PCR Amplification of Dumbbell Vector DNA

PCR amplification of the universal template and appendage of shRNA encoding DNA was carried out using 1 U *Taq* DNA polymerase (Invitrogen), 1.0 μM of each primer and blocking ODNs, 0.2 mM of each 2’-deoxyribonucleoside 5’-triphosphate (dNTP; Invitrogen), 100 ng of *Hind*III/*Bam*HI cleaved pVax1-UT, 5% v/v DMSO (Thermo Fisher Scientific, Waltham, MA, USA) in a reaction volume of 30–50 μL in 1× *Taq* DNA polymerase buffer (Invitrogen). Linearization of pVAX1-UT usually improves the PCR yields but is not essential. Thermal cycling was carried out as follows: initial denaturation at 96°C for 5 mins 27 cycles of denaturation (95°C, 30 s), annealing (59°C, 30 s), and extension (72°C, 1 min); and final extension at 72°C for 10 mins. A 50-μL PCR reaction yielded about 10 μg DNA.

#### Strand Separation and Annealing

PCR products were purified through silica-membrane-based spin columns (QIAquick PCR purification kit, QIAGEN, Hilden, Germany). Purified products were diluted to 400 μL in 1× hybridization buffer (1 M NaCl, 100 mM MgCl_2_, and 200 mM Tris-HCl, pH 7.4), heat-denatured at 96°C for 5 min followed by gradual cooling to room temperature to allow for intramolecular folding of plus- and/or minus-strand dumbbell vectors. The resulting DNA was concentrated using ethanol precipitation, pelleted by centrifugation, and resuspended in nuclease-free water.

#### Ligation of Single-Stranded Loop DNA

1 to 6 μg (∼10 to 60 pmol) of DNA was incubated with 2.5 mM MnCl_2_, 1 M betaine (Sigma, St. Louis, MO, USA), and 50 to 100 U CircLigaseII ssDNA ligase (Epicenter, Madison, WI, USA) in 1× CircLigaseII reaction buffer at 60°C for 16 h, followed by heat inactivation of the ligase at 80°C for 10 min. Highest conversion yields were observed when ligating 6 μg DNA with 100 U CircLigase.

#### Exonuclease Treatment

After ligation, products were treated with 10 U of T7 DNA polymerase (Thermo Fisher Scientific, Waltham, MA, USA) at 37°C for 1 h followed by heat inactivation at 80°C for 10 min. Products were assessed on 10% native polyacrylamide gels or 1% agarose gels, stained with ethidium bromide post-electrophoresis, and/or purified using phenol-chloroform-isoamylalcohol (25:24:1) extraction (1×), chloroform-isoamylalcohol (24:1) re-extraction (3×), and ethanol precipitation.

### Target Gene Knockdown Assays

#### Luciferase Knockdown Assays

HEK293T cells were maintained in DMEM (Hyclone, South Logan, UT, USA) supplemented with 10% (v/v) fetal bovine serum (Hyclone, South Logan, UT, USA) and 1% penicillin-streptomycin antibiotic solution (Thermo Fisher Scientific, Waltham, MA, USA). 24 h prior to transfection, 2 × 10^4^ cells/well were seeded in a 96-well plate. Cells were co-transfected with 100 ng of luciferase expression plasmid pGL3 (Promega, Madison, WI, USA) and 1.5 pmol or 0.5 pmol of either plus- or minus-strand dumbbell vector DNA using Lipofectamine 2000 (Thermo Fisher Scientific, Waltham, MA, USA) and a reagent:DNA ratio of 1:2.5. For the positive control (pGL3 only), empty pVAX1 (Thermo Fisher Scientific, Waltham, MA, USA) was used as feeder DNA to ensure all cells received the same quantity of DNA. 48 h post-transfection, cells were washed with sterile PBS and lysed in 20 μL passive lysis buffer (Promega, Madison, WI, USA) for 20 min, employing gentle shaking. 10 μL of lysate was treated with 50 μL of LARII reagent (Promega, Madison, WI, USA), and luminescence was quantified on the Biotek reader (Biotek Instruments, Winooski, VT, USA).

#### Monitoring Lamin A/C Knockdown by Intracellular Fluorescence-Activated Cell Sorting (FACS)

HEK293T cells were cultivated and seeded in 96-well plates 24 h prior to transfection as described above. Cells were transfected with 0.1, 0.5, or 2.5 pmol dumbbell vector DNA or 3 pmol siGENOMELamin A/C control siRNA (Dharmacon, Lafayette, CO, USA) using Lipofectamine 3000 (Thermo Fisher Scientific, Waltham, MA, USA) according to the manufacturer’s protocol. Medium was changed 24 h post-transfection, and cells were harvested after 48 h. For FACS analyses, the media was aspirated, and the cells were rinsed once with PBS before trypsinization with 50 μL of 1× trypsin-EDTA (Gibco). Trypsinized cells were collected by centrifugation at 4,200 rpm for 6 min in 200 μL media. Pelleted cells were resuspended in 100 μL media, fixed and permeabilized with intracellular fixation and permeabilization buffer set (eBioscience, San Diego, CA, USA) according to manufacturer’s protocol prior to intracellular staining. To assess lamin A/C knockdown, cellular lamin A/C was stained by anti-lamin A+C antibody (ab133256) (1/200) and donkey anti-rabbit IgG H&Ls AF647 (ab150075) (1/200) (Abcam, Cambridge, UK). FACS was performed on LSRFortessa cell analyzer, and FACSDiva software v6.1.3 (BD Biosciences, San Jose, CA, USA) was used for the acquisition of the samples. FlowJo software V10.5.2 (Tree Star, Ashland, OR, USA) was used for data analyses.

### Computational Secondary Structure Prediction

Minimum free energy secondary structures of DNA and RNA were folded using the algorithms *mfold* and/or *RNAfold*.^17^, ^18^

### Statistical Analysis

Diagrams represent mean values ± SEM of three independent experiments. The statistical analysis was performed using repeated one-way ANOVA with Tukey’s post hoc multiple comparison’s test (luciferase knockdown data) or using Student’s t test (lamin A/C knockdown data). The GraphPad Prism version 6 software (GraphPad, La Jolla, CA, USA) was used for the statistical analysis. p values are as indicated.

## Author Contributions

V.P. developed the concept. V.P., S.L.C., A.G., P.S.L., and G.S.X.T. designed the experiments. S.L.C., A.G., P.S.L., and G.S.X.T. carried out the experiments, and all authors analyzed the data. V.P., S.L.C., A.G., and P.S.L. wrote the manuscript.

## Conflict of Interest

The authors declare no competing interests.

## Supplemental Information

Document S1. Figures S1–S6

Document S2. Article plus Supplemental Information

## References

[1] Biasco L., Baricordi C., Aiuti A. (2012). Retroviral integrations in gene therapy trials. Mol. Ther..

[2] Yin H., Kanasty R.L., Eltoukhy A.A., Vegas A.J., Dorkin J.R., Anderson D.G. (2014). Non-viral vectors for gene-based therapy. Nat. Rev. Genet..

[3] Mok P.L., Cheong S.K., Leong C.F., Chua K.H., Ainoon O. (2012). Extended and stable gene expression via nucleofection of MIDGE construct into adult human marrow mesenchymal stromal cells. Cytotechnology.

[4] Kaur T., Slavcev R.A., Wettig S.D. (2009). Addressing the challenge: current and future directions in ovarian cancer therapy. Curr. Gene Ther..

[5] López-Fuertes L., Pérez-Jiménez E., Vila-Coro A.J., Sack F., Moreno S., Konig S.A., Junghans C., Wittig B., Timón M., Esteban M. (2002). DNA vaccination with linear minimalistic (MIDGE) vectors confers protection against Leishmania major infection in mice. Vaccine.

[6] Schakowski F., Gorschlüter M., Junghans C., Schroff M., Buttgereit P., Ziske C., Schöttker B., König-Merediz S.A., Sauerbruch T., Wittig B., Schmidt-Wolf I.G. (2001). A novel minimal-size vector (MIDGE) improves transgene expression in colon carcinoma cells and avoids transfection of undesired DNA. Mol. Ther..

[7] Zanta M.A., Belguise-Valladier P., Behr J.-P. (1999). Gene delivery: a single nuclear localization signal peptide is sufficient to carry DNA to the cell nucleus. Proc. Natl. Acad. Sci. USA.

[8] Fogg J.M., Kolmakova N., Rees I., Magonov S., Hansma H., Perona J.J., Zechiedrich E.L. (2006). Exploring writhe in supercoiled minicircle DNA. J. Phys. Condens. Matter.

[9] Jiang X., Yu H., Teo C.R., Tan G.S., Goh S.C., Patel P., Chua Y.K., Hameed N.B., Bertoletti A., Patzel V. (2016). Advanced design of dumbbell-shaped genetic minimal vectors improves non-coding and coding RNA expression. Mol. Ther..

[10] Cost G.J. (2007). Enzymatic ligation assisted by nucleases: simultaneous ligation and digestion promote the ordered assembly of DNA. Nat. Protoc..

[11] Taki M., Kato Y., Miyagishi M., Takagi Y., Taira K. (2004). Small-interfering-RNA expression in cells based on an efficiently constructed dumbbell-shaped DNA. Angew. Chem. Int. Ed. Engl..

[12] Taki M., Kato Y., Miyagishi M., Takagi Y., Sano M., Taira K. (2003). A direct and efficient synthesis method for dumbell-shaped linear DNA using PCR in vitro. Nucleic Acids Res. Suppl..

[13] Jiang X., Patzel V. (2017). Formation of Minimised Hairpin Template-transcribing Dumbbell Vectors for Small RNA Expression. Bio. Protoc..

[14] Yu H., Jiang X., Tan K.T., Hang L., Patzel V. (2015). Efficient production of superior dumbbell-shaped DNA minimal vectors for small hairpin RNA expression. Nucleic Acids Res..

[15] Myslinski E., Amé J.C., Krol A., Carbon P. (2001). An unusually compact external promoter for RNA polymerase III transcription of the human H1RNA gene. Nucleic Acids Res..

[16] Zeng Y., Wagner E.J., Cullen B.R. (2002). Both natural and designed micro RNAs can inhibit the expression of cognate mRNAs when expressed in human cells. Mol. Cell.

[17] Zuker M. (2003). Mfold web server for nucleic acid folding and hybridization prediction. Nucleic Acids Res..

[18] Hofacker I.L. (2003). Vienna RNA secondary structure server. Nucleic Acids Res..

