# Supplementary material for: Universal Template-Assisted, Cloning-free Method for the Generation of Small RNA-Expressing Dumbbell-Shaped DNA Vectors
Source: Mol Ther Methods Clin Dev. 2019 Aug 31;15:149–56. doi: 10.1016/j.omtm.2019.08.008 (PMC6807298; doi:10.1016/j.omtm.2019.08.008)
Supplement: Document S1. Figures S1–S6 [file mmc1.pdf]

OMTM, Volume *15*

Supplemental Information

Universal Template-Assisted, Cloning-free Method for the Generation of Small RNA-Expressing Dumbbell-Shaped DNA Vectors

Samantha Leeanne Cyrill, Avantika Ghosh, Pei She Loh, Genim Siu Xian Tan, and Volker Patzel

Supplemental Information

Figure S1

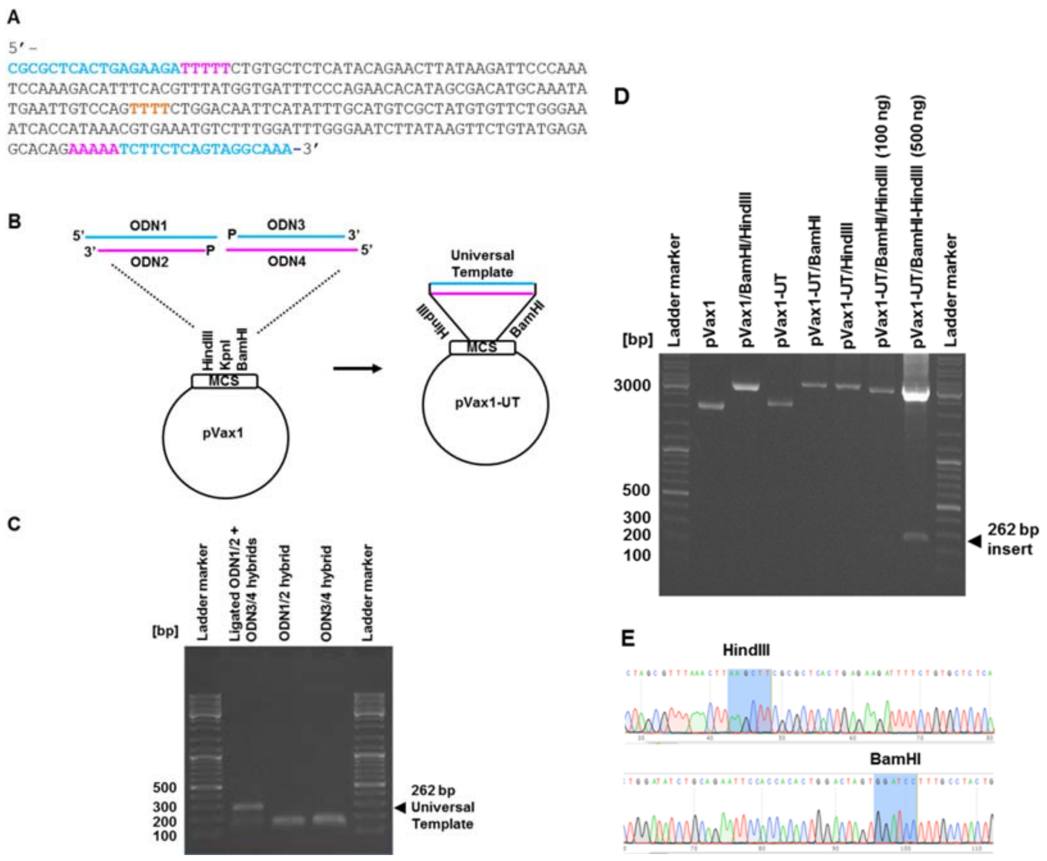

Figure S1. Sequence, cloning and validation of universal template (UT). (**A**), UT plus strand sequence. Cyan: miR-30 5' and 3' stem; magenta: transcriptional terminator (sense and antisense); orange: loop-forming tetranucleotide sequence; black: mH1 sequence (sense and antisense). (**B**), UT cloning scheme. 5' phosphorylatyed oligos ODN2 and ODN3 were annealed with oligos ODN1 and ODN4, respectively. The resulting duplexes were ligated and the double-stranded UT sequence was cloned into pVax1 using the *Hind*III and *Bam*HI restriction sites. Cyan: UT plus strand; magenta: UT minus strand. (**C**), Analytical 1% agarose gel electrophoresis showing the annealed oligo pairs ODN1/2 and ODN3/4 as well as the ligated hybrids. (**D**) Analytical *Hind*III and/or *Bam*HI digestion and 1% agarose gel electrophoresis of the cloned plasmid pVax1-UT. *Hind*III/*Bam*HI double digestion indicates the 262 bp UT band. (**E**), Sequencing of the *Hind*III and *Bam*HI cloning sites of pVAX1-UT.

Figure S2

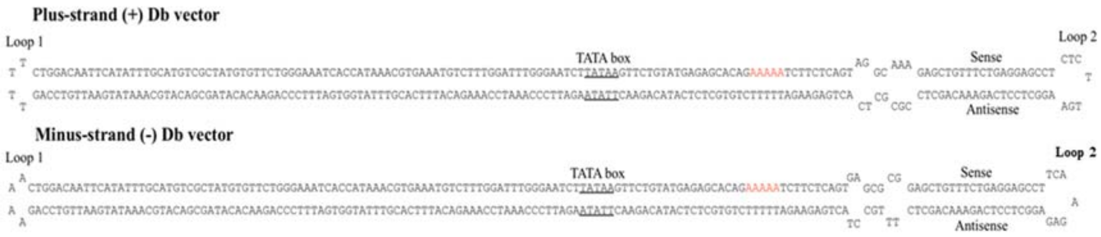

Figure S2. Sequences and structures of the complete plus and minus strand-derived luciferase-targeting dumbbell vectors. The lamin A/C-targeting vectors harbour the same dumbbell cores.

Figure S3

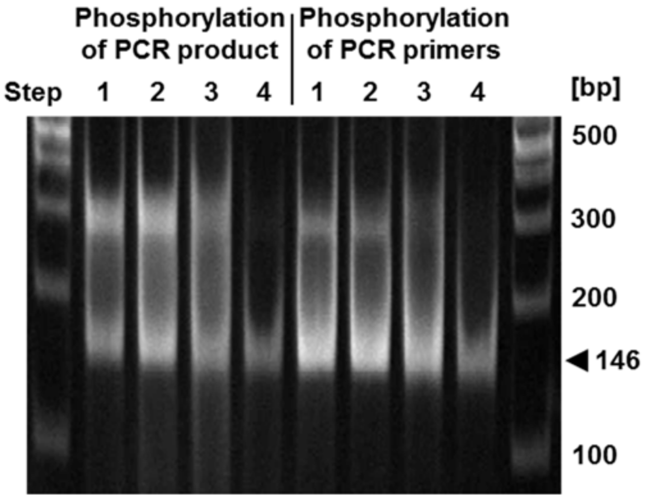

Figure S3. Dumbbell vector generation. Generation of 146 bp dumbbell vector DNA by 5' phosphorylation of the PCR product (left side) or alternatively by using 5' phosphorylated primers for the PCR reaction (right side). Steps are referring to Steps 1 to 4 defined in Figure 1.

Figure S4

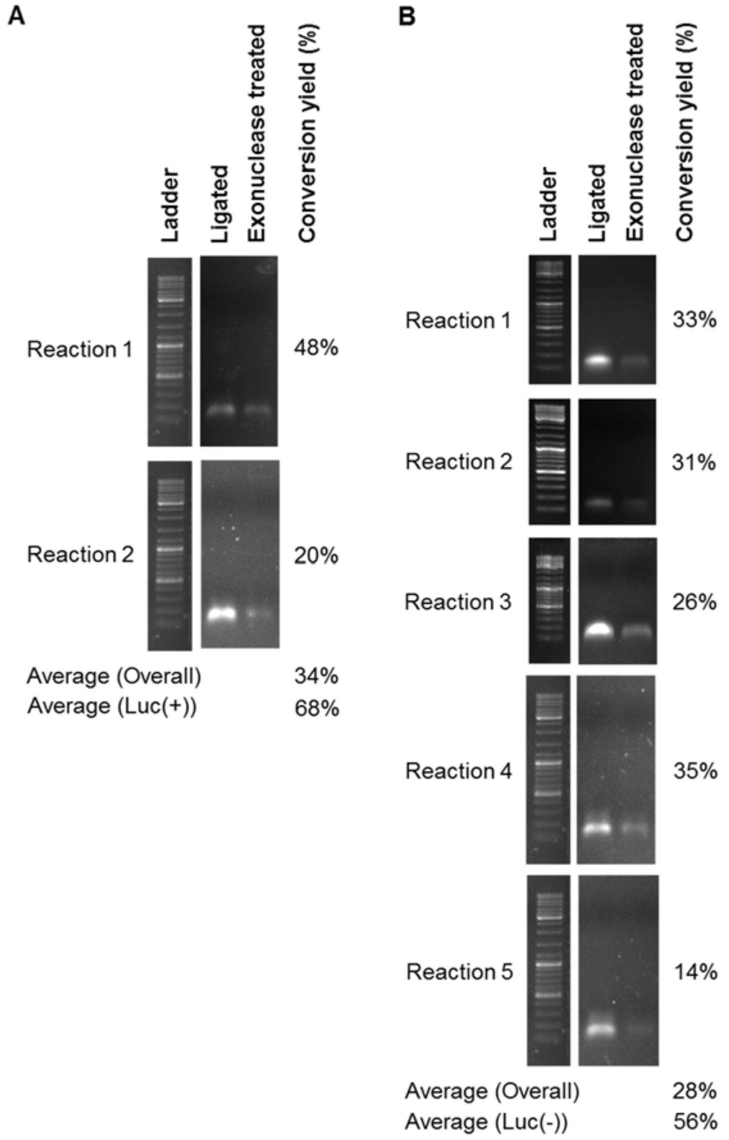

Figure S4. Determination of conversion yields for the plus (A) and minus (B) strand-derived luciferase-targeting dumbbells. PCR was performed using a non-phosphorylated forward and a 5'-phosphorylated reverse primer. 6 μg PCR products were denatured, refolded, and ligated in 50 μl reaction volume using 100 U CircLigase. Ligated refolded dumbbell DNA was treated with exonuclease and directly comparable samples before and after exonuclease were analysed using 1% agarose gel electrophoresis. Only successfully ligated covalently closed dumbbell DNA resists exonuclease treatment. Yields of converting non-ligated into ligated overall dumbbell DNA where determined by quantifying band intensities using the software ImageJ v1.48. Since either only the plus or the minus strand-derived dumbbell DNA, i.e. only 50% of the total DNA, can theoretically be ligated in each reaction, the actual conversion yield of plus or minus strand-derived dumbbell DNA is twice as high as the detected overall conversion yield.

Figure S5

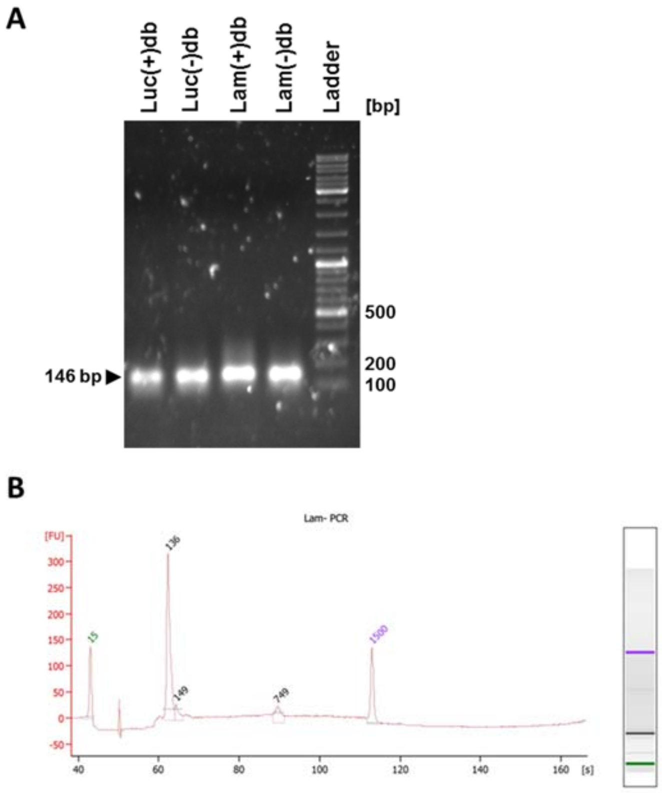

Overall Results for sample 8 : __Lam- PCR__

Number of peaks found: 3

Peak table for sample 8 : __Lam- PCR__

Peak		Size [bp]	Conc. [ng/µl]	Molarity [nmol/l]	Observations
1	◀	15	4.20	424.2	Lower Marker
2		136	10.47	116.7	
3		149	1.14	11.6	
4		749	1.04	2.1	
5	▶	1,500	2.10	2.1	Upper Marker

Figure S5. Purity control of dumbbell vectors after exonuclease treatment. (**A**), 1% agarose gel electrophoresis of plus (+) and minus (-) strand-derived luciferase (Luc) and lamin A/C (Lam)-targeting dumbbell (db) vectors. (**B**), Capillary gel electrophoresis (Bioanalyzer, Agilent) of Lam(-)db DNA. The identified 136 bp peak refers to the 146 bp dumbbell vector DNA. The difference in detected and expected vector size refers to the fact that the gel retardation of dumbbell vector DNA slightly differs from that of double-stranded DNA fragments of the DNA ladder marker.

Figure S6

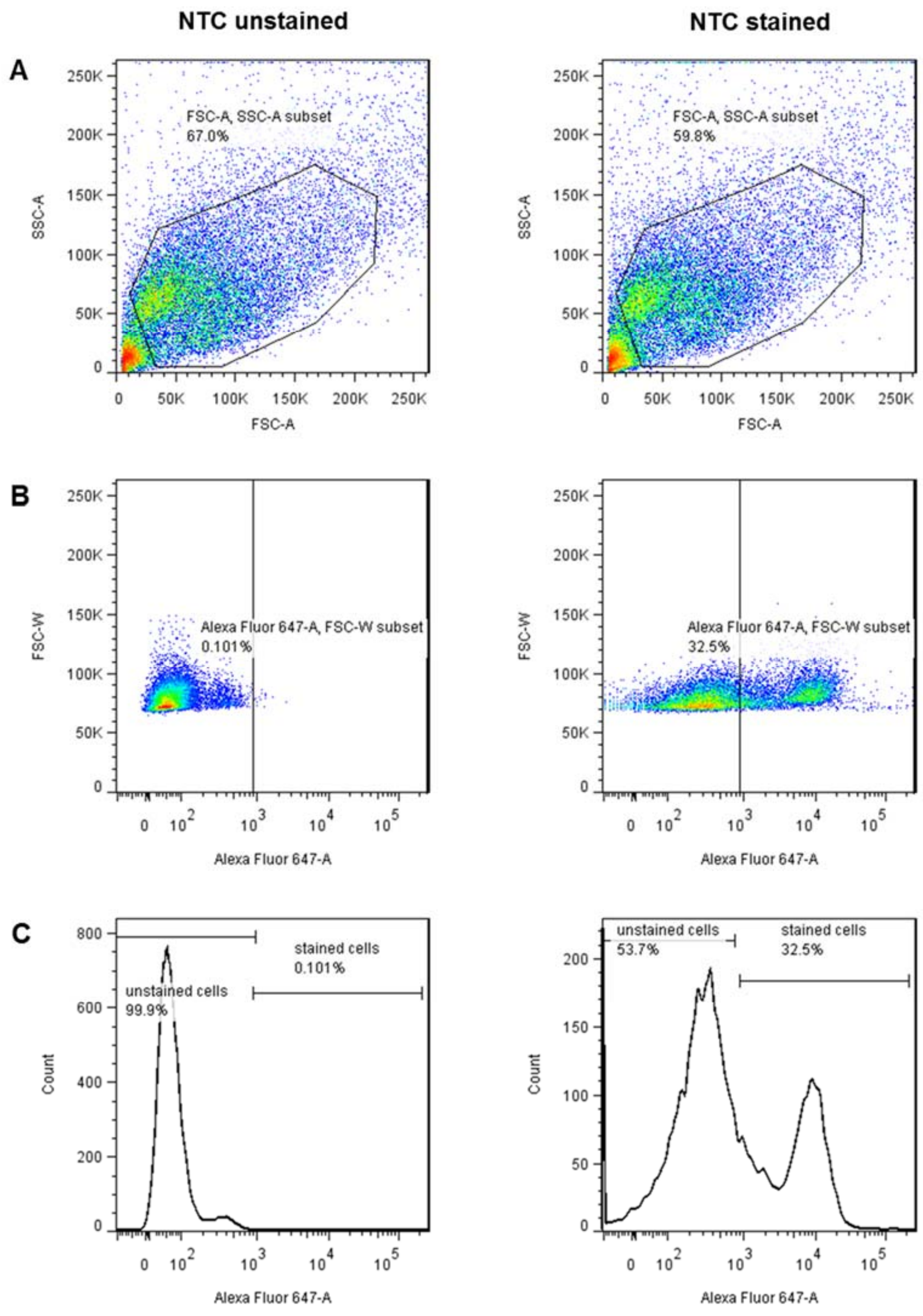

Figure S6. Monitoring lamin A/C expression by intracellular fluorescence-activated cell sorting (FACS). Cellular lamin A/C expression in non-transfected cells (NTC) was labelled by anti-lamin A+C primary antibody (left panels: unstained) and then stained with donkey anti-rabbit IgG H&L Alexa Fluor 647-A secondary antibody (right panels: stained). Gating of live HEK293T cells (**A**), Alexa Fluor 647-A scatter plots (**B**), and histograms (**C**).
